# Dogs as Reservoirs for *Leishmania donovani*, Bihar, India, 2018–2022

**DOI:** 10.3201/eid3012.240649

**Published:** 2024-12

**Authors:** Anurag Kumar Kushwaha, Ashish Shukla, Breanna M. Scorza, Rahul Chaubey, Dharmendra Kumar Maurya, Tulika Kumari Rai, Shyamali Yaduvanshi, Shweta Srivastava, Gaetano Oliva, Epke A. Le Rutte, Rajiv Kumar, Om Prakash Singh, Puja Tiwary, Shakti Kumar Singh, Scott A. Bernhardt, Phillip Lawyer, Edgar Rowton, Christine A. Petersen, Shyam Sundar

**Affiliations:** Banaras Hindu University, Varanasi, India (A.K. Kushwaha, A. Shukla, D.K. Maurya, T.K. Rai, S. Yaduvanshi, S. Srivastava, R. Kumar, O.P. Singh, P. Tiwary); University of Iowa, Iowa City, Iowa, USA (B.M. Scorza, C.A. Petersen); Kala-Azar Medical Research Center, Muzaffarpur, India (R. Chaubey, S. Sundar); University of Naples Federico II, Naples, Italy (G. Oliva), Swiss Tropical and Public Health Institute, Basel, Switzerland (E.A. Le Rutte); National Museum of Natural History, New Delhi, India (S.K. Singh); Utah State University, Logan, Utah, USA (S.A. Bernhardt); Walter Reed Army Institute for Research, Silver Spring, Maryland, USA (P. Lawyer, E. Rowton)

**Keywords:** Xenodiagnosis, dogs, parasites, leishmaniasis, vector-borne infections, *Leishmania donovani*, *Phlebotomus*, seasonal, transmission, zoonoses, global health, One Health, India

## Abstract

Visceral leishmaniasis derived from *Leishmania donovani* is transmitted by sand flies (*Phlebotomus argentipes*) throughout the Indian subcontinent*.* Although considered anthroponotic, *L. donovani* infects other mammals susceptible to sand fly bites, including dogs. Aggressive strategies to reduce sand fly populations in India have led to flies seeking nonhuman hosts, so understanding the role of dogs in *L. donovani* transmission has become critical. Our study investigated *L. donovani* infection in dogs and the potential for such infections to be transmitted back to sand flies. We performed xenodiagnosis by using *P. argentipes* on dogs (n = 73) with quantitative PCR–detectible parasitemia in both endemic and outbreak villages. We found that 12% (9/73) of dogs were infectious to sand flies during winter and rainy seasons. Patients with visceral leishmaniasis remain primary sources of *L. donovani* transmission, but our findings suggest a possible link between canine infection and human exposure.

Visceral leishmaniasis (VL), caused by *Leishmania donovani*, is transmitted by *Phlebotomus argentipes* on the Indian subcontinent. Depending on the geographic region of India, 2 different epidemiologic cycles sustain VL: a zoonotic cycle (*Leishmania infantum*), in which dogs are the primary reservoir, and an anthroponotic cycle (*L. donovani*). Active case detection and indoor residual spraying programs have reduced phlebotomine sand fly populations and have been linked to substantial declines in VL incidence across India (*1*). Although indoor residual spraying campaigns have made considerable progress in reducing VL in India, Nepal, and Bangladesh, and elimination was validated in Nepal and Bangladesh in 2020, India remains endemic and has persistent areas of infection in Uttar Pradesh and Bihar districts. The World Health Organization is formulating 2030 targets to identify knowledge gaps and sustain elimination of the disease, simulating interventional impact—including early outbreak identification—to impede or avoid VL spread (*2*,*3*). Both elimination goals and maintenance of validated elimination would be at risk if nonhuman sources of infection were found. Current elimination strategies in India do not target nonhuman sources of VL.

We theorized that indoor residual spraying might have altered *P. argentipes* behavior from endophilic to exophilic patterns. In fact, some reports have shown *P. argentipes* to feed indiscriminately on multiple mammalian hosts, particularly cattle and dogs (*4*,*5*). However, cattle, goats, and buffalo were not hosts for *L. donovani* (*6*). Although the role of dogs in *L. donovani* transmission remains unclear, canine reservoirs are well accepted as a major source of zoonotic transmission to maintain periurban and rural *L. infantum* infection (*7*). On the Indian subcontinent, health officials reconsidered nonhuman transmission of *Leishmania* spp. parasites after detection of *Leishmania* antibodies and *Leishmania*-specific DNA was demonstrated from livestock (*8*,*9*). *L. donovani* DNA was detected in the blood of village dogs, goats, and cows from India and Bangladesh, suggesting *L. donovani* infection (*8*–*11*). We investigated the potential role of dogs in the ecology of *L. donovani* on the Indian subcontinent, particularly whether dogs were capable hosts for transmission of *L. donovani* parasites to *P. argentipes* sand flies.

## Materials and Methods

### Selection of Study Area and Epidemiologic Database

**Figure 1:**
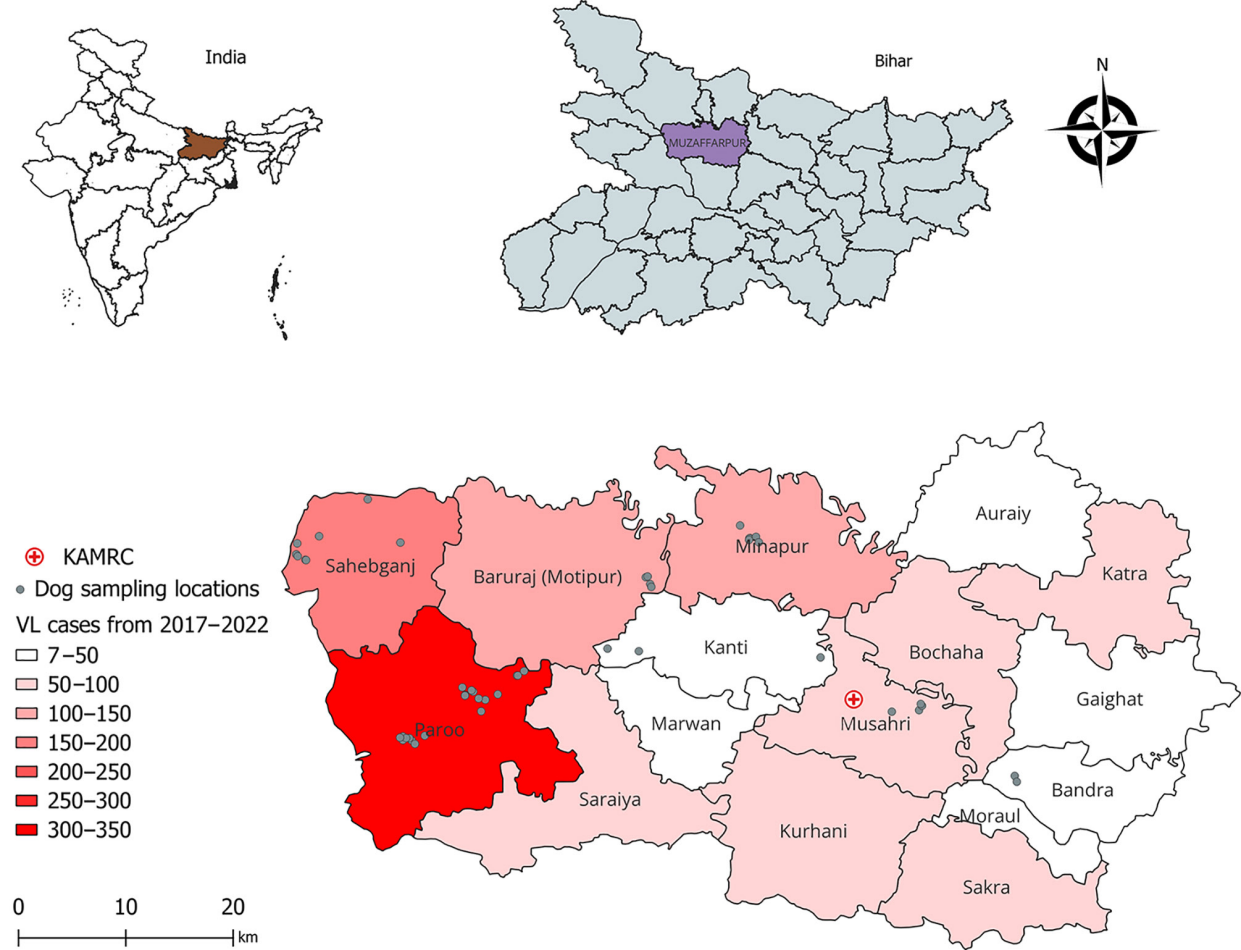
Locations of study villages and 5-year incidence of VL in a study of dogs as reservoirs for *Leishmania donovani*, Bihar, India, 2018–2022. A) Map of India showing Bihar; B) Bihar detail showing Muzaffarpur district study area; C) detail of sampling locations and VL cases, Muzaffarpur district. Geographic information system locations of study sampling sites calculated from a latitude-longitude application. Map produced using QGIS software version 3.30.3 (https://qgis.org) with open-access shapefile (https://onlinemaps.surveyofindia.gov.in). KAMRC, Kala-Azar Medical Research Center.

This study included 15 villages in the Muzaffarpur district (26.07°N, 85.45°E) of the state of Bihar, India, with endemic VL (*12*). Sampled villages had active *Leishmania* transmission during 2017–2022. Temperatures in Muzaffarpur range from 14°C during December–January to 32°C during April–May, and the average annual precipitation is ≈1,300 mm during the monsoon season during late June–September. We selected villages with active transmission based on VL history determined by the Health and Demographic Surveillance System (*13*) and Kala-azar Management Information System (*14*) (Figure 1).

### Sampling Procedures

We found that in endemic villages, dogs roamed near households without shelter, exposing them to harsh weather, and dogs often were malnourished. We surveilled the canine population and captured 10% of dogs across seasons. We captured mixed-breed male and female dogs, typically 1–4 years of age, based on physical appearance of illness and requested recruiters to seek out “sick” dogs for the study. We anesthetized dogs intravenously with Dexdomitor (Zoetis Inc., https://www.zoetisus.com) and reversed anesthetic effects with Antesedan (Zoetis Inc.), according to estimated bodyweight. We obtained physical exams and history, when available. We assessed disease state based on physical signs of canine leishmaniosis (CanL), including lymphadenopathy, anemia, dermatitis, and rough hair coat (*15*), and scored each dog on a 5-scale basis (i.e., number of clinical signs). We administered rabies vaccine to all dogs sampled. We collected blood samples in ethylenediaminetetraacetic acid vials and m-tube vials, transported samples on ice packs, and then aliquoted and stored samples at −20°C for serologic and molecular assays (Appendix Figure 1). We performed xenodiagnosis by using laboratory-reared *P. argentipes* sand flies on 73 dogs from 15 villages in the Muzaffarpur district.

### Preparation of Sand Flies for Xenodiagnosis

We used *P. argentipes* sand flies from a closed, certified, pathogen-free colony for xenodiagnoses, as previously described (*16*,*17*). We loaded 30–35 mature (3- to 5-days-old), 12-hours-starved *P. argentipes* female flies plus 10 males into feeding cups.

### Xenodiagnosis on Dogs

We assessed infectiousness of dogs in endemic villages by direct feeding of *P*. *argentipes* on the animals (xenodiagnosis) (*6*,*7*). We placed feeding cups on the ears and inguinal areas of sedated dogs for 30 minutes. We performed xenodiagnosis under local environmental conditions. We transferred blood-engorged females to 1-pint cups and kept them in an environmental chamber at 27°C and 80% humidity for 48 hours with access to a 30% sugar solution. We stored flies in 70% ethanol for processing.

### DNA Extraction from Whole Blood and Blood-Fed Sand Flies

We extracted DNA from whole blood by using QIAamp DNA Blood Mini Kit (QIAGEN, http://www.qiagen.com), according to manufacturer’s instructions. We extracted DNA from individual blood-fed sand flies by using Gentra Puregene Tissue DNA Extraction Kit (QIAGEN), optimized for individual sand flies (*7*) and endemic site (*6*). We assessed DNA quality by using Nanodrop Spectrophotometer (Thermo Scientific, https://www.thermofisher.com). We used DNA samples with 260/280 ratio 1.8–2.0 and 260/230 ratio >1.5 for real-time quantitative PCR (qPCR).

### qPCR

We performed quantification of parasites in whole blood and blood-fed flies by qPCR. We ran TaqMan-based qPCR on each DNA sample in duplicate on an Applied Biosystems 7500 Real-Time PCR system (Thermo Fisher Scientific) to amplify an *L. donovani* kinetoplast minicircle kDNA4 target with forward primer (4GGGTGCAGAAATCCCGTTCA), reverse primer (4 CCCGGCCCTATTTTACACCA), and probe (ACCCCCAGTTTCCCGCCCCG) (*6*,*17*). We used nuclease-free water (Thermo Fisher Scientific) and blood DNA from nonendemic healthy control dogs and DNA from pooled uninfected laboratory-reared *P. argentipes* as negative controls. We calculated quantification of parasite equivalents in test samples by using a standard curve generated from DNA from healthy human blood and uninfected sand flies spiked with a serial dilution of cultured *Leishmania* spp. parasites run in parallel to each set of test samples, as previously described (*18*). We considered PCR cycle threshold cutoff to be >35 for negative for blood and tissue. For sand flies, we considered a stringent cycle threshold >30 to be xenodiagnosis negative (*6*,*7*).

### Recombinant K39 Antigen ELISA

To measure antibodies in dog serum against recombinant K-39 (rK39) antigen, we coated 25 ng/well of rK39 antigen to 96-well, flat-bottom microtiter plates in coating buffer (0.1 mol carbonate-bicarbonate buffer, pH 9.6) and incubated samples overnight at 4°C. We blocked the plates with blocking buffer (1% bovine serum albumin in 0.05 mol phosphate buffer) at 25°C for 2 hours. We then added 100 μL of serum samples (1:200 dilution) to the plates and incubated plates at 25°C for 30 minutes. We assayed each sample in duplicate. We washed the plates with phosphate-buffered saline (pH 7.4) containing 0.1% Tween 20 (Sigma-Aldrich, https://www.sigmaaldrich.com). We used rabbit anti-dog IgG peroxidase conjugated secondary antibody (1:4,000 dilution, Sigma-Aldrich) with o-phenylenediamine dihydrochloride for 15 minutes and measured optical density at 490 nm (*7*). We used serum from infected dogs with CanL as assay-positive controls (C.A. Petersen, unpub. data).

### Statistical Analyses

We determined cutoff values for positive serology by adding 2 standard deviations to the mean optical density of canine-negative control sera (C.A. Petersen, unpub. data). We determined *Leishmania* spp. exposure prevalence based on rK39 ELISA. We assessed normality of data by using the D’Agostino-Pearson test. For comparisons between subjects or groups, we performed Mann-Whitney test or Fisher exact test. When appropriate, we used Kruskal-Wallis with Dunn’s posttest for multiple comparisons.

## Results

In *L. infantum*–endemic areas with vector transmission, 67%–80% of dogs had *Leishmania* antibodies or were positive for *Leishmania* DNA by qPCR, but characteristic of the ratio of asymptomatic to symptomatic disease, some dogs that had no outward clinical signs of CanL were infectious to sand flies (*19*). As we reported in a prior study, domestic cattle, goats, buffalo, and rodents were exposed to *L. donovani* parasites as evidenced by seropositivity on rK39 ELISA but did not show evidence of clinical infection and were not infectious to sand flies (*6*). To establish whether dogs had active infection or disease after exposure to *L. donovani*, we used physical and clinicopathologic examination, reinforced by diagnostic parameters, to evaluate dogs for their clinical status in an *L. donovani*–endemic area in Bihar, India. We analyzed parasitemia by qPCR and *Leishmania* serology by using blood from village dogs. We included 73 dogs in this study (Table). 

**Table:** Canine diagnostic test outcomes and sand fly qPCR positivity after xenodiagnosis in a study of dogs as reservoirs for *Leishmania donovani*, Bihar, India, 2018–2022*

Clinical signs	Diagnostic test results	No. CanL-positive dogs/no. dogs				No. qPCR-positive fed sand flies/no. dogs		
		Summer	Rainy	Winter		Summer	Rainy	Winter
Total no. (%), n = 73	NA	16 (22)	47 (64)	10 (14)		16 (22)	47 (64)	10 (14)
No CanL clinical signs, n = 44	qPCR–, ELISA–	7/9	17/30	5/5		0	5/2	1/1
	qPCR+, ELISA–	0	2/30	0		0	0	0
	qPCR–, ELISA+	2/9	9/30	0		0	0	0
	qPCR+, ELISA+	0	2/30	0		0	0	0
CanL clinical signs, n = 29	qPCR–, ELISA–	2/7	0	3/5		0	0	9/2
	qPCR+, ELISA–	0	2/17	0		0	0	0
	qPCR–, ELISA+	5/7	11/17	2/5		0	9/2	8/2
	qPCR+, ELISA+	0	4/17	0		0	0	0

*CanL, canine leishmaniosis; NA, not applicable; qPCR, quantitative PCR; +, positive; –, negative.

**Figure 2:**
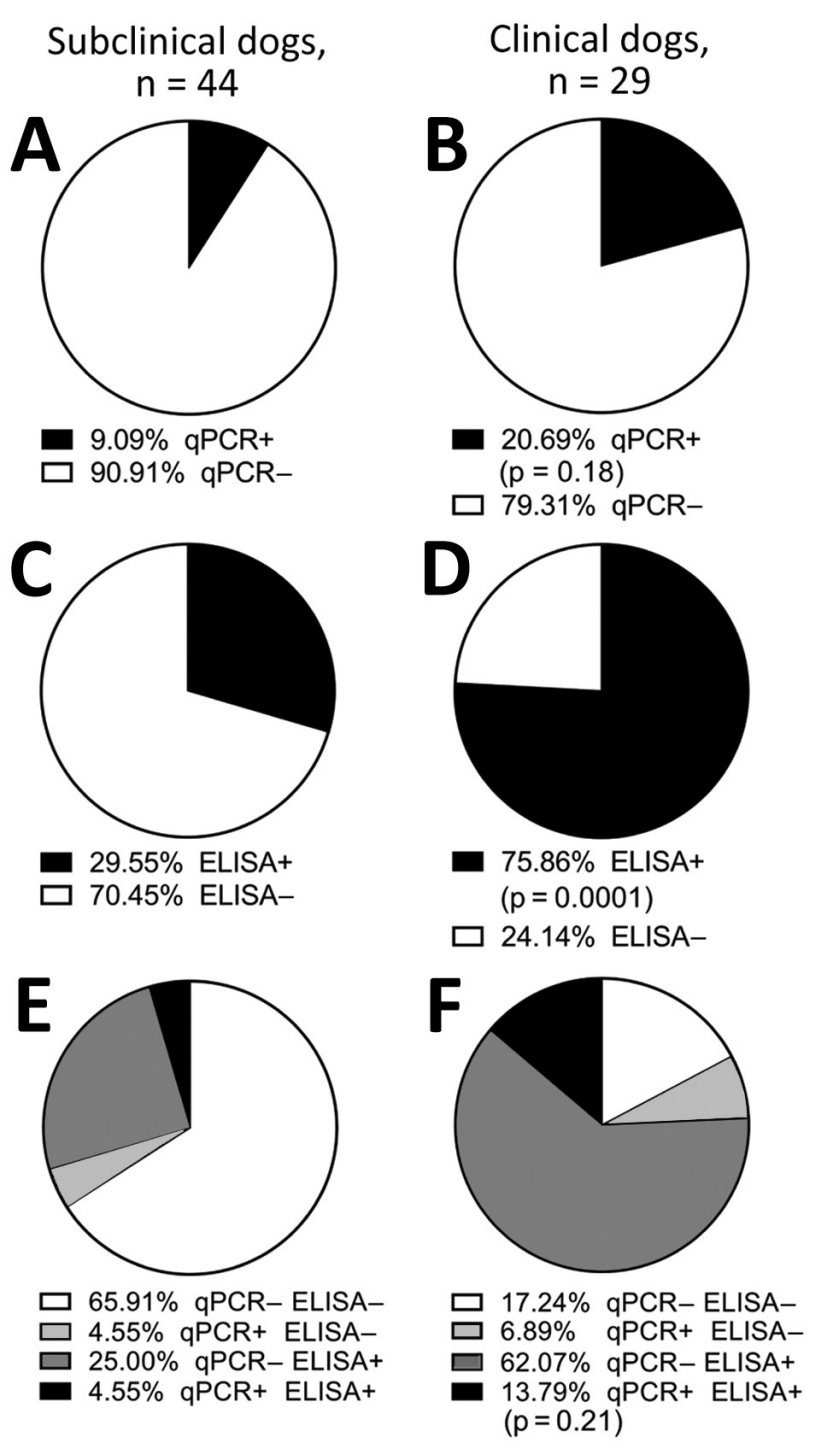
Seropositivity among endemic dogs with clinical signs consistent with CanL from the Muzaffarpur district in a study of dogs as reservoirs for *Leishmania donovani*, Bihar, India, 2018–2022. A,C,E) Results from subclinical (healthy) dogs; B,D,F) results from dogs with >2 clinical signs of CanL. Percentage of positive (black) versus negative (white) results by kinetoplastid-targeted qPCR or by rK39 ELISA are shown. E,F) Percentage of dogs with single-positive or double-positive diagnostic status among subclinical dogs (E) and clinical dogs (F). Statistical significance between subclinical and clinical groups measured by Fisher exact test. CanL, canine leishmaniosis; qPCR, quantitative PCR; +, positive; −, negative.

Despite asking for “sick dogs,” we classified 60% (44/73) of the dogs as subclinical and the other 40% (29/73) as clinical (i.e., >2 physical signs of CanL). Among subclinical dogs, 9% (4/44) were *Leishmania*-positive by blood qPCR (Figure 2, panel A). We determined that 21% (6/29) of clinically affected dogs had detectable parasitemia (Figure 2, panel B) (Fisher exact test p = 0.18). Thirty percent (13/44) of subclinical dogs had *Leishmania* antibodies via rK39 ELISA (Figure 2, panel C), and 76% (22/29) of dogs with clinical signs consistent with CanL were seropositive for *Leishmania* antibodies (Figure 2, panel D) (Fisher exact test p = 0.0001). Only 5% (2/44) of subclinical dogs were qPCR and ELISA positive (Figure 2, panel E); 14% (4/29) of clinical dogs were positive by both qPCR and rK39 ELISA (Figure 2, panel F) (Fisher exact test p = 0.21). Dogs from VL-endemic villages in Bihar with clinical signs consistent with CanL were >2 times more likely to have been exposed to *Leishmania* parasites as demonstrated by rK39 ELISA and 3 times more likely to have either been exposed or demonstrated parasitemia with *L. donovani* than other dogs from the same villages.

**Figure 3:**
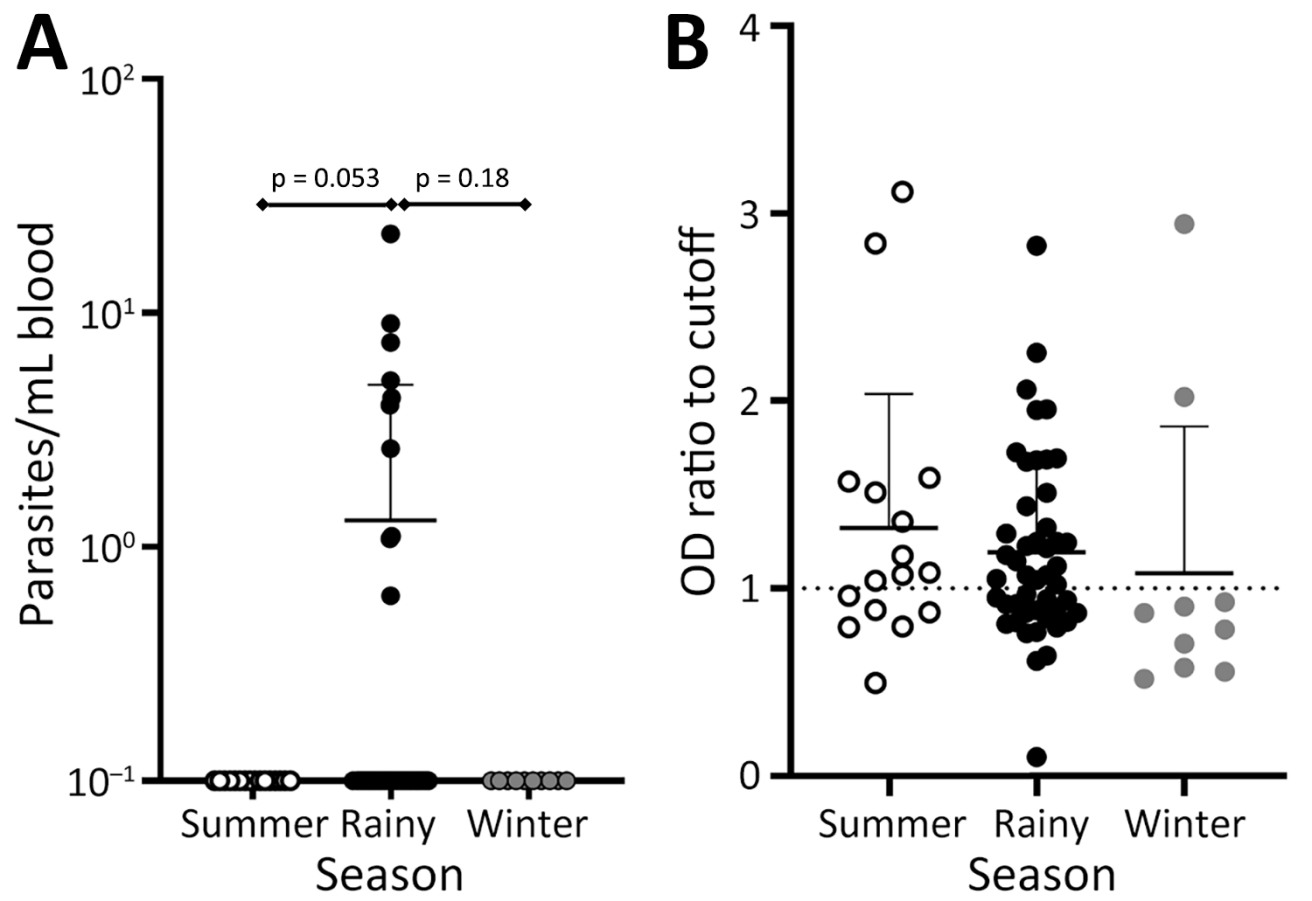
Plot of dogs with detectable parasitemia during the rainy season from villages in the Muzaffarpur district in a study of dogs as reservoirs for *Leishmania donovani*, Bihar, India, 2018–2022. Lower level of whiskers indicate mean, upper level indicates standard deviation. A) Parasitemia measured by quantitative PCR of whole blood DNA. Statistical significance between different seasons (shown above diamond bars) measured by Fisher exact test. B) *Leishmania* rK39 ELISA absorbance ratio detected from canine serum. Dotted line indicates OD cutoff. Kruskal-Wallis with Dunn’s posttest was performed, but no statistically significant differences were found for B. OD, optical density.

Temperature and rainfall (humidity) can play a crucial role in vector emergence and survival, directly affecting transmission of *Leishmania* spp. (*15*). Bihar has 3 distinct seasons with different temperatures and humidity. Research has documented seasonal differences in human VL incidence, and most transmission occurs in the rainy season (*20*). To better understand the timing of *L. donovani* parasite transmission to and from dogs, we assessed both parasitemia level and rK39 antibody production in dogs across seasons: summer (n = 16), rainy (n = 47), and winter (n = 10) (Table). We found qPCR-confirmed *L. donovani*–positive dogs during only the rainy season. The difference between the rainy season and winter was not statistically significant (Fisher exact test p = 0.18), but the difference between the rainy season and summer was significant (Fisher exact test p = 0.05) (Figure 3). By comparison, we found no seasonal pattern for *Leishmania* rK39 antibody levels in endemic-village dogs.

**Figure 4:**
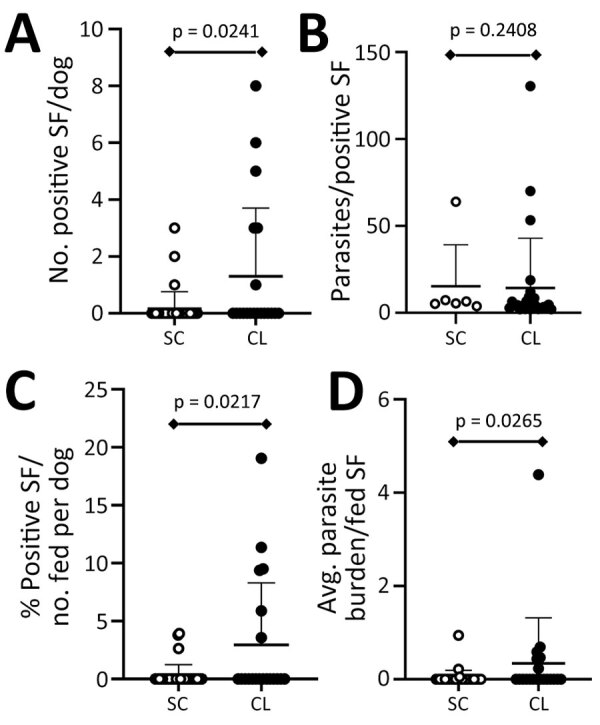
Plot of parasite load in sand flies in a study of dogs as reservoirs for *Leishmania donovani*, Bihar, India, 2018–2022. Plots show higher transmission and parasite load in sand flies fed on dogs with signs consistent with CanL in the Muzaffarpur district. A) Number of parasite DNA–positive sand flies obtained from each dog undergoing xenodiagnosis by qPCR. B) Parasite load calculated within individual *L. donovani* qPCR-positive sand flies. C) Percent positive sand flies out of total number of sand flies fed per dog. D) Average parasite burden of blood-fed sand flies per canine subject. Data for healthy, subclinical dogs depicted with white dots. Data for dogs with CanL clinical signs depicted with black dots. Lower lever of whiskers indicate mean, upper level indicates standard deviation. Statistical results by Mann-Whitney test shown above diamond bars. CanL, canine leishmaniosis; CL, CanL clinical signs; SC, subclinical (i.e., healthy) dogs; SF, sand fly.

*L. infantum*–infected dogs transmit parasites from skin to naive sand flies, and some evidence shows that clinically apparent dogs with anemia can be infectious (*7*,*19*,*21*). Popular understanding of *L. donovani* qualifies the species as anthroponotic and no previous evidence indicates dogs as a parasite source to *P. argentipes* sand flies. In performing xenodiagnosis on 73 dogs across villages endemic for VL (Figure 1), we found positive results from dogs with clinical signs consistent with leishmaniosis. Dogs with clinically observed signs of disease (CanL) provided a significantly higher average number of *Leishmania*-positive sand flies per dog than from dogs with <2 clinical signs of disease (p = 0.02) (Figure 4, panel A,B). A significantly higher percentage of sand flies fed on dogs with clinical signs consistent with CanL took up parasites (mean ≈3% of all fed sand flies) compared with those fed on subclinical dogs (mean ≈0.3% of fed sand flies, p = 0.02) (Figure 4, panel B,C). The average parasite burden among flies containing a blood-meal after feeding on dogs with clinical signs of CanL was significantly higher than the comparative average burden of parasites from blood-fed flies after xenodiagnosis on dogs without physical abnormalities (p = 0.03) (Figure 4, panel D). That finding is within the range of findings from patients with VL in previous studies (*22*,*23*).

We found that dogs showing signs of CanL had a higher rate of seroreactivity to *Leishmania* rK39 antigen, indicating previous exposure to the parasite. Dogs with signs of disease were better able to transmit parasites to sand flies. Pathogenesis of CanL and VL is widely believed to be secondary to antigen or antibody complexes and can be correlated with *Leishmania* antibody serologic levels (*24*). On the basis of this assumed correlation, we evaluated whether transmission from Bihar village dogs to sand flies would correlate with the presence of positive rK39 ELISA. We found that seropositive and seronegative dogs were similarly infectious to sand flies (Appendix Figure 2, panel A). We observed no significant difference in parasite uptake, percent positivity of sand flies, or average parasite burden within sand flies fed on rK39-seropositive versus rK39-seronegative dogs, indicating that parasite exposure (i.e., rK39 seropositivity) did not predict infectiousness to sand flies.

**Figure 5:**
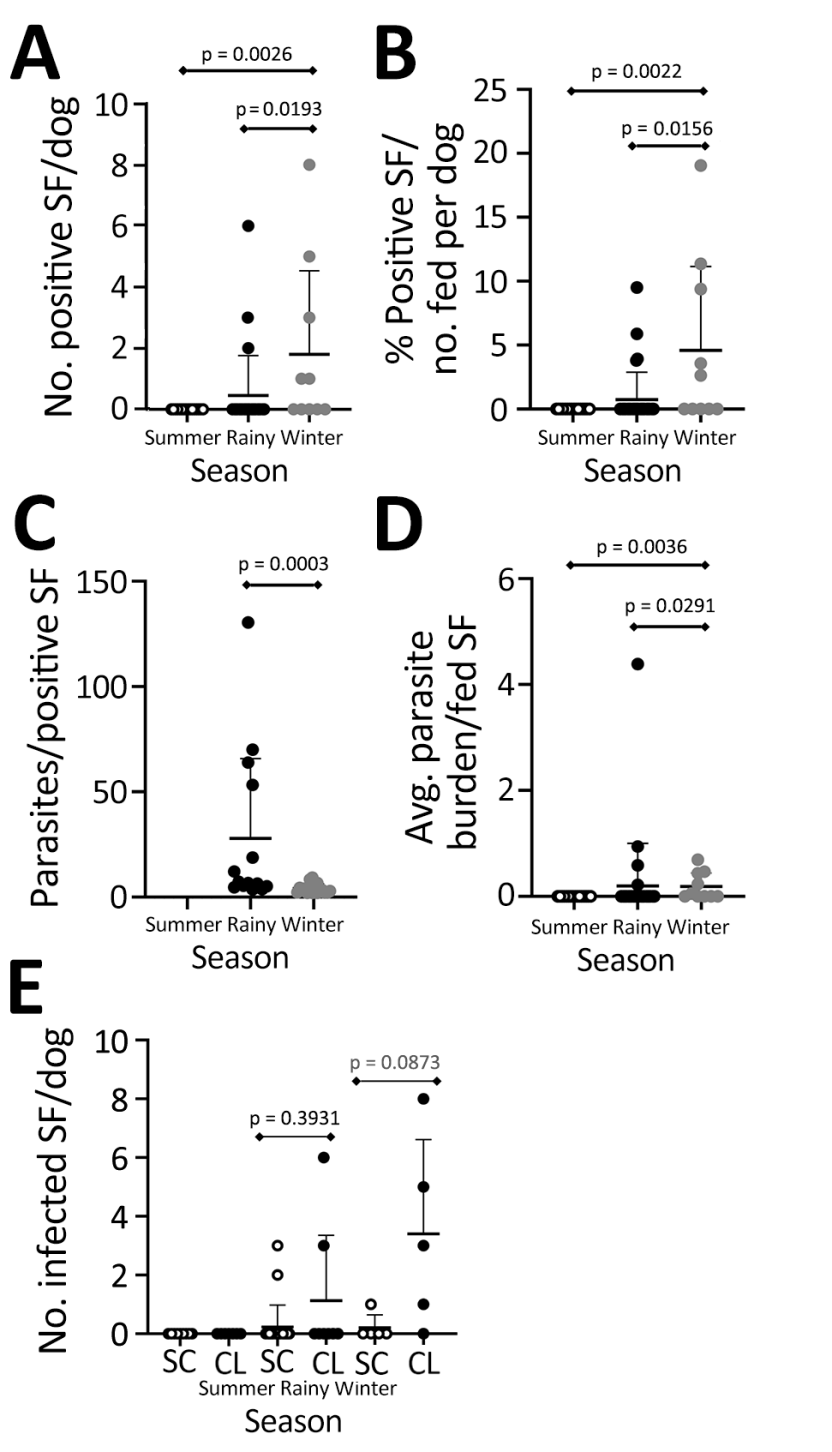
Results of xenodiagnoses in a study of dogs as reservoirs for *Leishmania donovani*, Bihar, India, 2018–2022. Plots show dogs infect more sand flies in winter and transmit more parasites per sand fly during the rainy season. A) Number of positive sand flies; B) parasite load in xenodiagnosis-positive sand flies; C) percent positive sand flies fed on dogs; D) average parasite burden of fed sand flies; E) evaluation of infectiousness of dogs to sand flies by clinical classification and seasonal variation. Lower lever of whiskers indicate mean, upper level indicates standard deviation. Kruskal-Wallis test with Dunn’s post-test used to calculate statistical significance between groups (shown above diamond bars). CanL, canine leishmaniosis; CL, CanL clinical signs; SC, subclinical (i.e., healthy) dogs; SF, sand fly.

Sand flies emerge during the rainy season, after the dry, hot summer, hungry and looking for blood meals (*20*). We found that dogs had higher parasitemia during the rainy season. Therefore, we wanted to test whether canine infectiousness was influenced by season. Sand flies that fed on dogs during rainy and winter seasons were positive for *L. donovani* kDNA as confirmed by qPCR (Figure 5). No dogs were infectious to sand flies via xenodiagnosis during summer (Figure 5, panel A). The percentage of sand flies positive for *Leishmania* DNA was highest during winter (Figure 5, panel B). Per dog, the average parasite burden in fed sand flies was higher in winter than in the rainy season (Figure 5, panel D). We noted that 50% (5/10) of parasite-positive sand flies were fed during winter, a rate higher than that for sand flies fed on dogs in the rainy season, 13% (4/30). Across both the rainy and winter seasons, 46% (6/13) of dogs with clinical signs were infectious to sand flies and only 11% (3/37) of subclinical dogs were infectious to sand flies (Figure 5, panel E). Our data revealed that canine infectiousness was associated with clinical disease, regardless of clinical classification or how infection was detected.

## Discussion

We investigated canine exposure to *L. donovani* parasites in Bihar, India, and performed xenodiagnosis to explore the role of dogs in transmitting parasites to sand flies. Published literature regarding *L. donovani* parasites suggests that the species is strictly anthroponotic on the Indian subcontinent, but studies have reported seropositivity in nonhuman mammals (*6*). *Leishmania* spp. are a parasite of sand flies, which serve as vessels where parasite meiosis occurs (*25*). Sand fly blood meal analyses in Bihar and elsewhere revealed that 10%–25% of sand flies took blood from dogs (*5*). Although controversial, the fact that dogs can be infected by and be a source of *L. donovani* parasites may not be surprising based on the ecology of *L. donovani*–complex parasites globally.

In our study, prevalence of *L. donovani* parasite exposure was high (45%, 35/73) in endemic dogs, consistent with other serologic studies of dogs (*9*,*11*). Sand fly abundance is highly influenced by seasonality. Previous studies showed that sand flies emerge and are most abundant during the rainy season (*20*), and that the highest proportion of gravid females and highest parasitic loads were found in summer (*20*,*26*). We found the highest canine seropositivity in summer, perhaps due to significantly more parasitized sand flies and gravid females feeding in preparation of egg laying.

Quantitative serology is a sensitive tool for CanL surveillance and positive serology has previously been correlated with presence of clinical signs (*27*). We found that 76% (22/29) of dogs with signs consistent with CanL were seropositive for *L. donovani* antibodies. Our analyses revealed that 55% (5/9) of seronegative dogs were infectious to *P. argentipes* sand flies, a finding consistent with previous study results showing that 27.4% of seronegative dogs had *L. infantum* infection (*28*). We did not observe a correlation between the serostatus of dogs and their relative infectiousness to *P. argentipes* sand flies. Dogs with subclinical infections typically remain healthy for many years due to effective cell-mediated immunity (*7*). We performed molecular diagnostic tests to detect *Leishmania* DNA in dog blood. We detected low parasitemia from dogs during the rainy season. Conversely, we could not detect *Leishmania* DNA in dog blood from summer or winter. *L. infantum* DNA has been shown to rise over time in dogs, depending on disease presentation, bone marrow parasite load, and renal disease severity, often impacted by other comorbid diseases (*7*,*21*). Only after parasite burden increases in the bone marrow or dogs have advanced renal disease do they become consistently parasitemic. (*7*,*21*) Previous studies have revealed that after peak *Leishmania* parasite transmission season, because of the presence of many infectious sand flies, parasite DNA could be present in domestic mammals in proximity to humans (*6*).

Detection of PCR positivity does not dictate that dogs are critical for the life cycle of *L. donovani* parasites in India. Sand flies are telmophages that feed after skin laceration, so dermal parasite burden might be an important factor. Xenodiagnosis studies on patients with post-kala-azar dermal leishmaniasis (PKDL, a dermal leishmaniasis that manifests after VL as macular, papular, or nodular rash that usually appears on face, upper arms, and trunk [*2*]) revealed that parasitemia was very low, and no significant correlation was observed between dermal parasitic load and parasitemia. (*17*) Despite low parasitemia, 88% (23/26) of patients with PKDL transmitted parasites to at least 1 sand fly (*17*). Parasitemia, therefore, is not always the best predictor of a host’s outward transmission (*29*). Research has demonstrated *L. infantum* parasites to be especially dermotrophic, and skin-parasite burden is highly correlated with parasite transmission to sand flies (*19*). 

In our study, the proportion of dogs that transmitted *L. donovani* parasites to at least 1 sand fly was 12% (9/73), much lower than transmitted by dogs with active CanL due to *L. infantum* (58%, 15/26) (*30*). Our 12% finding is also lower than results from more recent xenodiagnosis studies in patients with nodular (67%, 18/27) and macular (35%, 9/27) PKDL and in patients with VL (67%, 10/15) in Bangladesh (*22*). In contrast, none of the 184 asymptomatic enrolled participants in a study population in Bihar, India were infectious to sand flies (*17*). Our data revealed sand flies were positive for kDNA qPCR after being fed on subclinical dogs (n = 3), but with a low resultant sand fly parasite load. Despite that finding, we theorize that low infectiousness of multiple village dogs could affect *L. donovani* parasite transmission and potentially be an outbreak source, particularly if canine parasitologic status remains stable and the number of individual infected dogs accumulates over time.

Our xenodiagnosis investigation showed that most fed sand flies acquired <100 parasites. Another study showed that a subsequent blood meal greatly augmented sand fly infectiousness (*31*), observing that sand flies acquired a larger burden (>100) of *Leishmania* amastigotes from a second feeding. In our experiments, we assessed only the presence of *L. donovani* parasites during the early stages of development in the vector 48 hours after 1 experimental blood meal. A relatively small number of parasites acquired by sand flies after feeding on infected dogs might be able to survive and replicate in the gut of the vector after a second blood meal. The *Leishmania* life cycle within the sand fly takes ≈8–10 days to reach stationary phase growth. Extending the post-xenodiagnosis time for parasite replication to the metacyclic stage would verify whether parasites can then be transmitted to humans. Such verification requires additional studies to understand outgoing sand fly infectiousness and the effect of >1 blood meal on infectious dogs.

Xenodiagnosis on livestock and rodents in endemic villages of Bihar indicated that those animals were exposed to *Leishmania* parasites but had a limited or no role in the spread of infection (*6*). Presence of rK39 antibodies with supporting *Leishmania* DNA–specific PCR from blood and blood-fed sand flies suggests that even though multiple domestic animal species are exposed to *L. donovani* after infectious sand fly bites, only dogs, known to be noteworthy reservoir species for other *L. donovani* complex spp., were infectious to sand flies. Our data suggest that, unlike livestock or rodents, dogs are infectious to sand flies and present a risk for outbreak infections in areas where human disease elimination has been established in India. Dogs have been implicated as a bridge between the sylvatic cycle of *Leishmania* to persons. A study of the emergence of *L. infantum–*based CanL in Israel indicated a high prevalence of infected dogs, in the presence of a competent vector species, which led to the onset of parasite transmission to humans in the area (*1*). 

In conclusion, identifying and establishing the role of dogs in the ecology of *L. donovani* by investigating the extent to which they contribute to disease transmission is critical. Increased understanding of a causal link between infected dogs and humans—or vice versa (reverse zoonoses)—can be garnered through additional epidemiologic studies. Research has shown effective prevention of parasite transmission from dogs to sand flies through application of topical insecticides or insecticide-impregnated collars (*32*). Outbreak villages serve as ideal settings for natural experiments to assess topical insecticide interventions focused on preventing transmission from infected dogs and sustaining the elimination efforts in India and wherever *L. donovani* is endemic. Health officials should consider topical or oral insecticidal interventions that prevent sand fly feeding on dogs in epidemic villages to maintain elimination.

Appendix Additional information on dogs as reservoirs for *Leishmania donovani*, Bihar, India, 2018–2022.
