# Supplementary material for: Dogs as Reservoirs for Leishmania donovani, Bihar, India, 2018–2022
Source: Emerg Infect Dis. 2024 Dec;30(12):2604–13. doi: 10.3201/eid3012.240649 (PMC11616638; doi:10.3201/eid3012.240649)
Supplement: Appendix — Additional information on dogs as reservoirs for Leishmania donovani, Bihar, India, 2018–2022. [file 24-0649-Techapp-s1.pdf]

Article DOI: http://doi.org/10.3201/eid3012.240649

EID cannot ensure accessibility for supplementary materials supplied by authors. Readers who have difficulty accessing supplementary content should contact the authors for assistance.

Dogs as Reservoirs for *Leishmania donovani*, Bihar, India, 2018–2022

Appendix

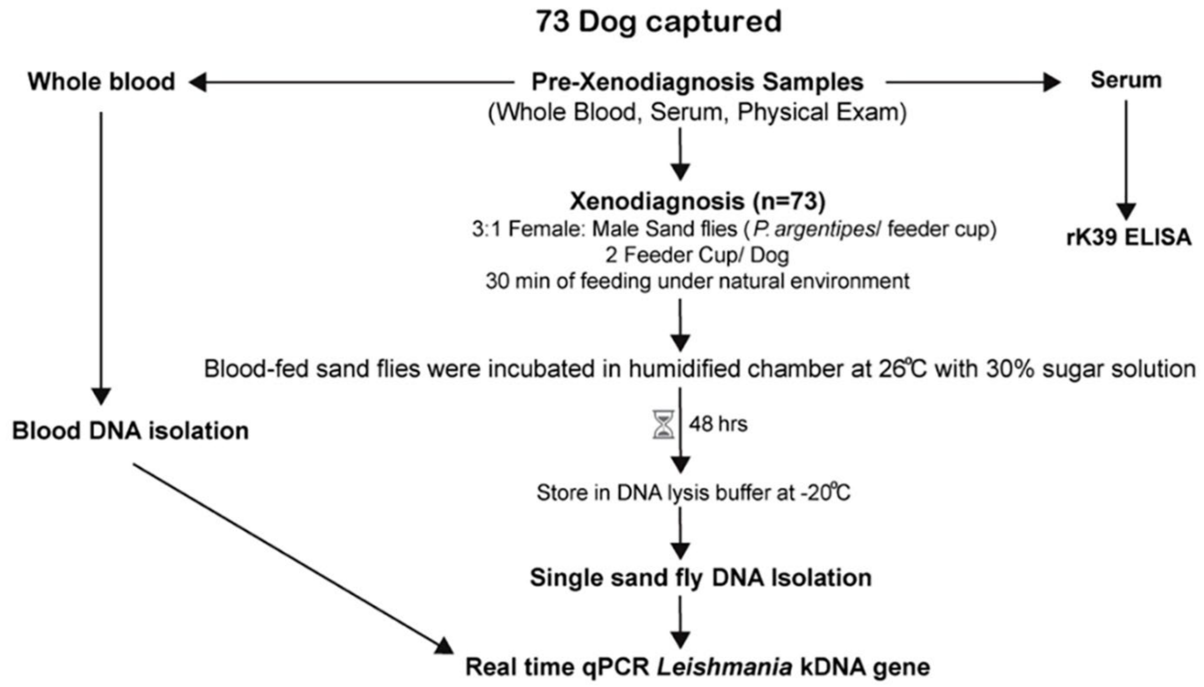

Appendix Figure 1. Diagrammatic representation of study design and sampling procedures for a study of dogs as reservoirs for *Leishmania donovani*, Bihar, India, 2018–2022.

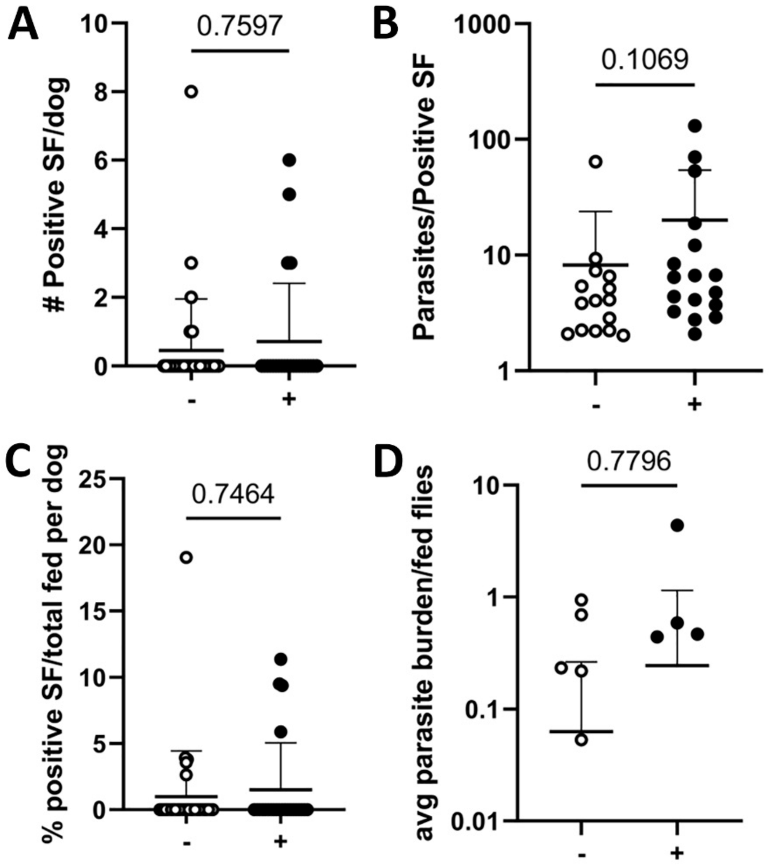

Appendix Figure 2. Canine serostatus was not associated with sand fly infectiousness based on study investigating dogs as reservoirs for *Leishmania donovani*, Bihar, India, 2018–2022. A) Number of sand flies (SF) containing parasites after feeding on rK39 ELISA seropositive (+) and seronegative (–) dogs. B) Parasite load calculated within individual *L. donovani* qPCR-positive SF. C) Percent positivity of SF fed on seropositive and seronegative dogs. D) Average parasite burden of fed flies measured via single SF qPCR. Mean +/− standard deviation is shown. Mann-Whitney test used for statistical analysis.
